# Cortical Tuning is Impaired After Perceptual Experience in Primary Visual Cortex of Serotonin Transporter-Deficient Mice

**DOI:** 10.1093/texcom/tgaa066

**Published:** 2020-09-16

**Authors:** Alexandr Pak, Alexander A Chubykin

**Affiliations:** Department of Biological Sciences, Purdue Institute for Integrative Neuroscience, Purdue University, West Lafayette, IN 47907, USA; Department of Biological Sciences, Purdue Institute for Integrative Neuroscience, Purdue University, West Lafayette, IN 47907, USA

**Keywords:** familiarity, oscillations, primary visual cortex, serotonin, visual experience

## Abstract

Serotonin (5-hydroxytryptamine) is crucial for the proper development of neuronal circuits early in life and their refinement throughout adulthood. Its signaling is tightly regulated by the serotonin transporter (SERT), alterations of which were implicated in various neurological and psychiatric disorders. Animal models lacking a functional SERT variant display diverse phenotypes, including increased anxiety, social communication deficits, and altered cortical development. However, it remains unclear how SERT disruption affects sensory processing and experience-dependent learning in adulthood. It has been previously shown that perceptual experience leads to the development of visual familiarity-evoked theta oscillations in mouse V1. Here, we discovered that familiarity-evoked theta oscillations were longer and less stimulus specific in SERT knockout (KO) compared with wild-type (WT) mice. Interestingly, while the overall visual response properties were similar in naive mice, orientation and spatial frequency processing were significantly impaired in SERT KO compared with WT or SERT heterozygous mice following perceptual experience. Our findings shed more light on the mechanism of familiarity-evoked oscillations and highlight the importance of serotonin signaling in perceptual learning.

## Introduction

The serotonergic system is involved in reward/punishment processing, behavioral inhibition, mood, depression, cognitive flexibility, learning, and memory (Sora et al. 1998; Lanfumey et al. 2000; Ansorge et al. 2004; Cohen et al. 2015; Matias et al. 2017; Lottem et al. 2018). Mutations in serotonin transporter (SERT) are implicated in various neurological and neuropsychiatric disorders. Mouse models with partial or full loss of SERT functionality displayed a plethora of phenotypes ranging from anxiety to altered cortical development (Bengel et al. 1998; Lira et al. 2003; Murphy and Lesch 2008).

Previous research suggests that 5-hydroxytryptamine (5-HT) plays a vital role in the sensory cortex. 5-HT is important for the remodeling of cortical circuits during development and adulthood. Visual cortex requires both sensory and neuromodulatory inputs, especially during early life, for proper development (Kojic et al. 2000; Gu 2002). Neuromodulators have been shown to regulate cortical plasticity and sensory processing during the critical period of development and adulthood (Wang et al. 1997). Consistent with these observations, an SERT inhibitor, fluoxetine, can reopen the critical period in the adult visual cortex allowing for plasticity to reoccur. This effect was mediated through reduced intercortical inhibition and increased brain-derived neurotrophic factor levels (Maya Vetencourt et al. 2008).

5-HT can also directly modulate cortical circuits during adulthood (Puig et al. 2004; Celada et al. 2013). Recent evidence suggests that activation of dorsal raphe serotonergic neurons inhibits baseline activity but not odor-evoked responses in the olfactory cortex (Lottem et al. 2016). Furthermore, another study in SERT-deficient rats described altered sensory processing in the somatosensory cortex mediated by the reduced feed-forward inhibition in layer IV of the barrel cortex, which subsequently altered sensory integration (Miceli et al. 2017). These findings suggest that 5-HT alterations might lead to altered sensory processing. Previous studies demonstrated the direct effects of 5-HT receptor agonists in rodent visual cortex. Fast spiking and low-threshold interneurons were shown to be modulated by 5-HT_3_ and 5-HT_1A_ receptor agonists in rat visual cortex slices (Xiang and Prince 2003). Another recent study showed that 5-HT_2A_ receptor agonist decreased visual processing and altered surround suppression in mouse V1 (Michaiel et al. 2019).

Previous experience has also been shown to alter the information processing in the primary visual cortex (V1). Presentations of phase-reversing gratings over several days lead to the increase in the amplitude of visually evoked potentials (VEPs), the phenomenon known as stimulus response potentiation (Frenkel et al. 2006; Cooke et al. 2015). Similarly, presentations of a sequence of sinusoidal gratings also lead to the potentiation of VEPs specific for the familiar sequence (Gavornik and Bear 2014). Repetitive pairings of a visual stimulus to a water reward delivered at a temporal delay lead to the development of a persistent neuronal activity, which can encode the time of the reward (Shuler and Bear 2006). This reward timing was dependent on the cholinergic muscarinic receptors (Chubykin et al. 2013). Interestingly, the persistent activity encoding reward timing has been shown to be in the form of persistent theta oscillation lasting to the time of reward (Zold and Hussain Shuler 2015). We have recently demonstrated that persistent theta oscillations could encode general visual stimulus familiarity without any reward presentation (Kissinger et al. 2018). These familiarity-evoked theta oscillations were also dependent on the muscarinic receptors. They were also impaired in Fmr1 knockout (KO) mice, a model of Fragile X syndrome, the most common inherited form of intellectual disability and autism (Kissinger et al. 2020). In addition to induction of the persistent activity, perceptual learning has been demonstrated to improve stimulus selectivity in the adult visual cortex (Cooke and Bear 2010; Hua et al. 2010; Gilbert and Li 2012; Makino and Komiyama 2015; Poort et al. 2015). Such improvements are mediated through increased selectivity and sharper tuning to a trained stimulus, but not at the expense of overall cortical tuning (Jurjut et al. 2017). However, it remains unclear how perceptual learning can modify cortical tuning in the case of altered 5-HT signaling.

Using silicon probe recordings, we investigated the role of the serotonergic neuromodulation in the familiarity-evoked theta oscillations and in the experience-dependent changes in cortical tuning using SERT heterozygotes (HET) and KO mice (Bengel et al. 1998). We found that orientation, spatial frequency (SF) tuning, and contrast sensitivity are not altered in naive mutant mice. The perceptual experience did, however, impair cortical tuning in SERT-deficient mice, especially in KO mice, in a multitude of ways. First, familiarity-evoked theta oscillations were longer and less specific in SERT KO mice. Second, we observed decreased orientation selectivity and broadened tuning width in SERT KO after the perceptual experience. Third, low SF responses were increased in SERT KO. Fourth, both SERT KO and HET showed altered contrast sensitivity after perceptual learning. Overall, we found intact visual processing in naive mice but impaired cortical tuning after perceptual experience in SERT-deficient mice.

## Materials and Methods

### Mice

All procedures involving animal use were approved by the Purdue University Animal Care and Use Committee. SERT KO mice, B6.129 (Cg)-Slc6a4^tm1Kpl^/J, were acquired from the Jackson Laboratory (stock no. 008355). We bred SERT HET and HET mice to generate SERT KO, HET, and wild-type (WT) littermate controls. In total, 26 mice were used: 4 SERT littermate control WT (2 male and 2 female), 8 HET (4 male and 4 female), 10 KO (5 male and 5 female), and 4 male age-matched control WT C57BL/6 mice. Mice were group-housed on a 12-h light/dark cycle with ad libitum water and food access.

### Surgical Protocol

Animal surgical procedures were performed as previously described (Kissinger et al. 2018). Briefly, about 2-month-old mice were induced with 5% isoflurane and head-fixed to a motorized stereotaxic apparatus (Neurostar). Their body temperature was maintained using a heating pad, and they were kept at 1.5–2% isoflurane anesthesia. The ophthalmic ointment was applied to prevent eye drying. Next, we shaved and sterilized the skin above the skull. The skull was exposed to install a small head post and a reference pin. Neurostar software with an integrated mouse brain atlas was used to label V1 coordinates (from lambda AP 0.8 mm, LM: ±3.2 mm) with a black marker. To fix the head post and seal all exposed areas, we used Medical grade Metabond. After surgery, mice were monitored for at least 3 days for any signs of distress or infection. Animals were then habituated to a head-fixation apparatus for at least 4 days and a minimum of 90 min per day while sitting in front of the computer monitor and viewing a gray screen. On the recording day, a small craniotomy was made above V1 in one of the hemispheres under 1.5% isoflurane anesthesia. Mice were then transferred to the recording room and head-fixed to the apparatus for electrophysiological recordings.

### In Vivo Electrophysiology

All experiments were performed in awake head-fixed mice. After animals were moved to the recording room, 30 min was allowed for them to recover from anesthesia. A 64-channel silicon probe (Shobe et al. 2015) (channel separation: vertical 25 μm, horizontal 20 μm, 3 columns, 1.05 mm in length) was inserted to perform acute extracellular electrophysiology. Each animal underwent a maximum of 2 recording sessions (one per hemisphere). Data were acquired at 30 kHz using OpenEphys hardware and software. An Arduino board was used to synchronize data acquisition and visual stimulus presentations using transistor-transistor logic (TTL) communication. We used custom-written Python scripts in PsychoPy (Peirce 2009) to present visual stimuli and send the TTL signals. After each recording session, silicon probes were cleaned in a trypsin (2.5%) solution.

### Histology

After electrophysiological recordings, 100 mg/kg ketamine and 16 mg/kg xylazine solution were used to anesthetize animals. Mice were then perfused transcardially with 1X phosphate-buffered saline (PBS) solution followed by 4% paraformaldehyde (PFA). After decapitation, the brain was extracted and stored in PFA in a refrigerator. The brain was sliced the following day in 0.1-mm sections in PBS using a vibratome. Coronal slices were mounted on slides using n-propyl-gallate media and sealed with transparent nail polish. A light microscope (VWR) was used to image slices for the electrode track verification in V1.

### Visual Stimulation

All visual stimulations were designed and presented using an open-source Python software, PsychoPy (Peirce 2009). Visual stimuli were binocularly presented on a gamma calibrated LCD monitor (22' ViewSonic VX2252, 60 Hz), which was placed 17 cm in front of the mouse. The mean luminance of the monitor was 30 cd/m^2^. For perceptual experience, mice were presented with the same visual stimulus (30° drifting grating, contrast = 100%, temporal frequency = 2 Hz, SF = 0.04 cpd, duration = 0.4 s) for 4 days, 200 presentations a day with an interstimulus interval of 3–5 s. For orientation tuning experiments, we presented sinusoidal drifting gratings of 12 different directions. Stimuli were created with the following parameters: contrast = 100%, SF = 0.04 cpd, temporal frequency = 2 Hz, and duration = 0.5 s. There was a 3–5 s intertrial interval. To generate visual stimulations for an SF tuning, we performed spatial filtering of white noise (Kissinger et al. 2018). Specifically, we band-pass filtered white noise in different nonoverlapping SF bands. The procedure, and a Python code for SF filtering, were adapted from http://www.djmannion.net/psych_programming/vision/sf_filt/sf_filt.html. Overall, 6 different spatial frequencies were generated for SF tuning: 7.5E-3, 0.015, 0.03, 0.06, 0.12, and 0.24 cycles/degrees. We chose these frequencies based on previous studies and known SF tuning of mouse V1 neurons (Niell and Stryker 2008). The use of these stimuli for SF tuning has been verified in our previous study (Kissinger et al. 2018). The SF tuning sequence contained 6 different SF stimuli presented in a pseudorandom order, each with an equal probability of being presented. We used an intertrial interval of at least 4 s to prevent any adaptation. Furthermore, SF filtered stimuli were randomly generated on each trial to sample different receptive fields uniformly. This was mainly important for lower spatial frequencies. For contrast sensitivity experiments, we presented a 0° oriented (vertical) static grating at 5 different contrast levels: 6.25%, 12.5%, 25%, 50%, and 100%.

### LFP Analysis

Broadband electrophysiology traces were first downsampled to 1 kHz. Symmetric linear-phase finite impulse control filter (default parameters) was then used to remove 60 Hz cable noise (MNE Python library). Next, we identified layer IV responses by finding a channel with the strongest negative deflection in the first 100 ms after stimulus onset. Complex wavelet convolution was used to perform time–frequency decomposition. We designed 40 different wavelets across a logarithmic range of 2–80 Hz, with cycles ranging from 3 to 10. This gave us an optimal time–frequency precision trade-off. These wavelets were convolved with averaged local field potentials (LFP) traces and then averaged to produce power spectra heatmaps that were dB baseline normalized. To quantify a mean power within a particular band, we averaged responses within 1 s after the stimulus onset. A total of 6 different frequency bands were used: theta (4–8 Hz), alpha (8–12 Hz), beta (12–30 Hz), low gamma (30–50 Hz), and high gamma (50–80 Hz). We also extracted phases of the signal and then quantified an intertrial phase coherence (ITPC) by averaging complex vectors defined by those angles within 0.5 s after the stimulus onset:

$$ \mathrm{ITP}{\mathrm{C}}_{tf}=\left|\frac{1}{N}{\sum}_{r=1}^N{\mathrm{e}}^{i{k}_{tf}}\ \right| $$

where *N* is the number of trials, ${\mathrm{e}}^{ik}$ indicates a complex polar representation of the phase angle *k* at a specific frequency (*f*) and time point (*t*) (Cohen 2014). We did not quantify ITPC for frequencies above 40 Hz as it will require a larger number of trials and can be limited by the monitor refresh rate.

### Single-Unit Analysis

Spike detection and sorting along with manual curation of units were performed as previously described (Kissinger et al. 2018). Briefly, Kilosort was used for spike detection and sorting (Pachitariu et al. 2016). Default configuration parameters were used for clustering, but a threshold for spike detection was changed from −4 to −6 SD. Templates were initialized from the data. Kilosort was run using MATLAB (Mathworks) on a computer running Windows 10. For clustering purposes, all the different recording sessions were concatenated together. This allowed us to track single neurons across different recording sessions performed on the same day. After spike detection and sorting, we visualized and verified clustering results using the Klusta/Phy graphical user interface, which was then used for manually removing, splitting, and merging units when necessary (Rossant et al. 2016). We only included high-quality single units that had a clear refractory period and a high-amplitude waveform template. To merge and split units, we followed the guidelines available online (https://github.com/kwikteam/phy-contrib/blob/master/docs/template-gui.md). Peristimulus time histograms (PSTHs) of single units were constructed by binning spike times across trials with 10-ms bins and convolving the obtained histogram with a Gaussian Kernel (width = 100 ms). The *z*-score was calculated using the following formula:

$$ z=\frac{\mathrm{FR}-\mathrm{mean}\left(\mathrm{baseFR}\right)}{\mathrm{sd}\ \left(\mathrm{baseFR}\right)} $$

where FR is a firing rate at each time point, and base refers to the baseline activity over 0–0.3 s for tuning experiments or 0–0.5 for all other recordings. To investigate the oscillatory activity, we focused on the neurons that upregulate their firing in response to visual stimuli. We used the Wilcoxon signed-rank test to identify these neurons by comparing baseline firing rate 0.05–0.5 s versus stimulus window 0.5–0.95 s. The oscillatory duration was quantified using a peak detection algorithm. The time of the last peak exceeding a 1.5 *z*-score firing rate was computed for each unit and stimulus condition. For orientation tuning experiments, the orientation selectivity index (OSI) was computed using the following formula (Ringach et al. 2002; Scholl et al. 2013):

$$ \mathrm{OSI}=\frac{\sqrt{\left(\sum{r}_k\sin \left(2{\theta}_k\right)\right){}^2+{\left(\sum{r}_k\cos \left(2{\theta}_k\right)\right)}^2\kern0.5em }}{\sum{r}_k} $$

where θ and *k* represent orientation (in radians) and stimulus index, respectively. This is a more robust measure of selectivity compared with a conventional measure. We also fitted a double Gaussian to find the tuning width sigma (σ):

$$ R\left(\theta \right)={R}_0+{R}_p{\mathrm{e}}^{\frac{-{\left(\theta -{\theta}_p\right)}^2}{2{\sigma}^2}}+{R}_n{\mathrm{e}}^{\frac{-{\left(\theta -{\theta}_p+180\right)}^2}{2{\sigma}^2}} $$

The function has 5 parameters to fit: baseline firing rate ${R}_0$, preferred orientation ${\theta}_{\mathrm{pref}}$, the response at the preferred orientation ${R}_p$, the response at the null orientation ${R}_n$, and tuning width σ.

For SF analysis, we first defined a preferred SF as the one that induces the strongest response (peak in the tuning curve). Population tuning curves were then constructed using normalized firing rates across different neurons. We also quantified a low SF suppression (LSFS) by dividing the response to the lowest SF tested (7.5E-3 cpd) by the response at the preferred SF. To quantify tuning bandwidth, we fitted a difference of Gaussian (DoG) function to SF tuning curves (Hawken and Parker 1987):

$$ R\left(\mathrm{SF}\right)={R}_0+{K}_e{\mathrm{e}}^{\frac{-{\left(\mathrm{SF}-{\mu}_e\right)}^2}{2{\sigma}_e^2}}-{K}_i{\mathrm{e}}^{\frac{-{\left(\mathrm{SF}-{\mu}_i\right)}^2}{2{\sigma}_i^2}} $$

This function has 7 free parameters: baseline firing rate ${R}_0$, amplitude ${K}_e$ and ${K}_i$, center ${\mu}_e$ and ${\mu}_i$, and width ${\sigma}_e$ and ${\sigma}_i$ of the excitatory and inhibitory components, respectively.

For contrast sensitivity curves, we fitted a hyperbolic ratio function (Albrecht and Hamilton 1982):

$$ R(c)={R}_0+{R}_{\mathrm{max}}\frac{c^n}{c_{50}^n+{c}^n} $$

where *c* is the contrast of the stimulus. It has 4 parameters: baseline firing rate ${R}_0$, maximum response ${R}_{\mathrm{max}}$, exponent *n*, and semisaturation point *c*_50_.

All curve fitting procedures were performed using the least-squares method in Python (scipy.optimize.curve_fit). The fitting error was defined as

$$ \mathrm{fit}\ \mathrm{error}=\frac{\sum{\left({y}_i-{f}_i\right)}^2}{\sum{\left({y}_i-\bar{y} \right)}^2} $$

where ${y}_i$ is the observed value, $\bar{y}$ is the mean of observed data, and ${f}_i$ is the fitted value. For statistical analysis of parameters, we only included units with a fitting error <0.7.

Population neural decoding for orientation, SF, and contrast responses were performed using linear discriminant analysis (LDA) in Python’s scikit-learn package (default parameters) (Virtanen et al. 2020). Population spike counts within 0.05–0.5 s relative to the stimulus onset were used to train classifiers. A 4-fold cross-validation with 5 repeats was performed. The number of folds was chosen so that the test size was not below 30 samples. The number of units used for training was comparable between pre and post perceptual experience and across different genotypes.

### Pupillometry

The detailed procedure has been previously described (Kissinger et al. 2018). Briefly, video acquisition of the mouse pupil was performed under infrared illumination. Videos were then analyzed post hoc using a Python computer vision library, OpenCV. We first performed a histogram equalization to enhance the contrast of the video frames. Manual thresholding was then used to detect putative pupil. Given a good preprocessing pipeline, we performed the pupil tracking by first detecting contours and then fitting a minimum enclosing circle. This ensured that whiskers and small local contrast variations did not affect the tracking. The *x*,*y*-coordinate and radius were extracted based on the fitted circle. We analyzed both a raw diameter of the pupil and area % change from the baseline. A subset of videos was analyzed with a DeepLabCut (Mathis et al. 2018). We trained a convolutional neural network (ResNet50) on a graphics processing unit (GPU) to detect pupil coordinates. In total, 250 frames from different mice and lighting conditions were used for training. The circle was fitted to 4 pupil coordinates using least-square optimization in Python. For validation purposes, we generated at least one labeled video for each mouse before including its data in the final analysis. Furthermore, we excluded outlier data points by thresholding pupil diameter to be in the range of 10–50 pixels.

### Statistical Analysis

Python’s scipy.stats library was used to perform all statistical analyses (Virtanen et al. 2020). We did not test the normality of residuals, and only nonparametric tests were used. Kruskal–Wallis test was used as a nonparametric version of analysis of variance when more than 2 groups were compared. A Mann–Whitney *U* test was used for pairwise comparisons. It was used to compare trial averaged LFPs, *z*-score firing rates, and pupil dynamics in different conditions and groups. It was also used to compare fitted parameters between genotypes.

**Figure 1:**
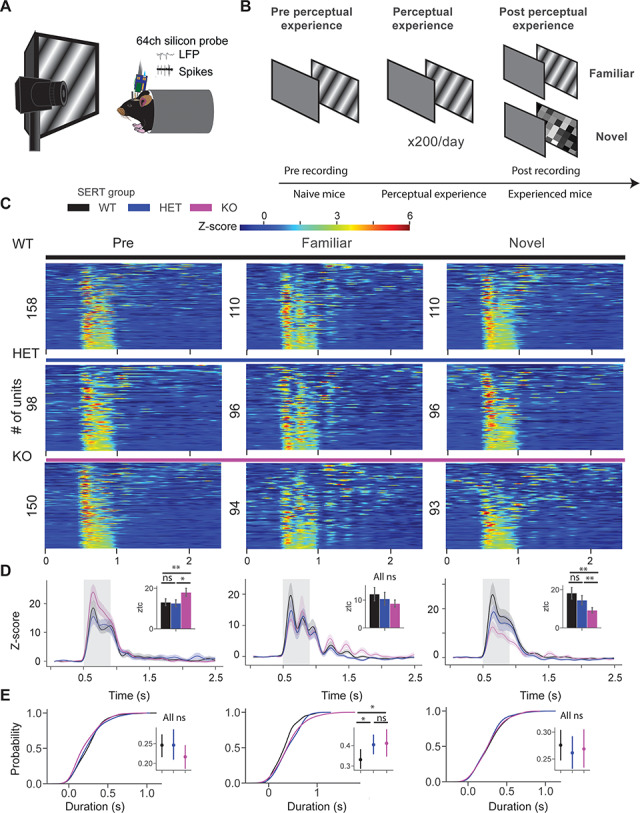
Longer visual experience-dependent oscillatory activity in units of SERT-deficient mice. (*A*) In vivo extracellular electrophysiology with 64-channel silicon probes in awake head-fixed mice. (*B*) Animals were recorded pre and post perceptual experience. During pre recording, a block of drifting grating stimuli (pre) was presented (×20, 0.5 s in duration). It was followed by tuning experiments, which consisted of orientation, spatial frequency, and contrast tuning experiments. During perceptual experience, a sinusoidal drifting grating was presented to animals 200 times a day for 4 days. Post recording was similar to pre, but animals were also presented with a novel stimulus, a checkerboard pattern (×20, 0.5 s). (*C*) Heatmap of the *z*-score firing rate of single units in pre (left), post familiar (middle), and post novel condition of SERT WT, HET, and KO mice. (*D*) Line plots show the mean of *z*-score responses of 3 different groups. Inset bar plots show the mean ± SEM of *z*-score within 0.05–0.5 s after the stimulus onset. (*E*) The cumulative distribution function of oscillatory duration across 3 different conditions. Inset point plots show the mean ± SEM of oscillatory duration across 3 genotypes.

## Results

### Increased Duration of Visual Experience-Dependent Oscillatory Activity in Neurons of SERT KO Mice

To investigate the role of 5-HT signaling in visual processing and experience-dependent learning, we performed in vivo silicon probe recordings using 64-channel probes in awake head-fixed SERT mice (Shobe et al. 2015) (Fig. 1*A*). We have previously shown that the perceptual experience of a sinusoidal drifting grating for 4 days (200 presentations per day) leads to the emergence of the theta oscillations specific to the familiar stimulus in mouse V1 (Kissinger et al. 2018), whereas the presentation of a novel stimulus did not evoke these oscillations. Using a similar paradigm, we investigated visual experience-dependent learning in SERT-deficient mice. A sinusoidal drifting grating (direction: 30°, SF: 0.04 cpd, temporal frequency: 2 Hz) was presented to mice 200 times a day for 4 days (Fig. 1*B*). Both electrophysiological and pupillometry recordings were performed before (pre) and after (post) perceptual experience. In line with our LFP findings and previous study, we observed oscillatory activity in single units after perceptual experience in all 3 groups (Fig. 1*C* and Supplementary Fig. 1). There was a significantly stronger grating-evoked population *z*-score response in naive SERT KO compared with other genotypes [Fig. 1*D* left, *z*-score firing rate pre: genotype WT vs. HET vs. KO (*P* = 0.01), Kruskal–Wallis test, *n* = 158, 98, and 150 units; WT vs. HET (*P* = 0.18), WT vs. KO (*P* = 0.003), and HET vs. KO (*P* = 0.03), post hoc Mann–Whitney *U* test]. There was no difference in *z*-score firing rate between groups in experienced mice [Fig. 1*D* middle, *z*-score firing rate post: genotype (*P* = 0.54), Kruskal–Wallis test, *n* = 110, 96, and 93 units]. Responses to the novel checkerboard stimulus were significantly weaker in SERT KO mice compared with other genotypes [Fig. 1*D* right, *z*-score firing rate novel: genotype (*P* = 0.004), Kruskal–Wallis test, *n* = 110, 96, and 93 units; WT vs. HET (*P* = 0.39), WT vs. KO (*P* = 0.001), and HET vs. KO (*P* = 0.004), post hoc Mann–Whitney *U* test]. Our pupillometry results were in line with our previous study. There was a strong surprise response (pupil dilation) to the visual stimulus in pre condition (naive mice), however, after perceptual learning, mice showed a surprise response in the novel but not in familiar condition. Baseline pupil size was qualitatively larger in SERT-deficient mice compared with WT, but it did not reach significance. However, a significantly weaker surprise response was observed in the novel condition in SERT KO compared with other groups (Supplementary Fig. 2). To quantify the duration of the oscillatory activity, we used a peak detection algorithm. The time point of the last detected peak in the PSTH of the unit was used as a measure of the duration of oscillations. There was no oscillatory activity in pre and novel condition, hence, no significant differences were observed [Fig. 1*E* left and right, duration pre: genotype (*P* = 0.21), Kruskal–Wallis test, *n* = 120, 80, and 125; novel: genotype (*P* = 0.82), Kruskal–Wallis test, *n* = 130, 128, and 81 units]. We observed a significantly longer oscillatory activity in experienced SERT-deficient mice compared with WT [Fig. 1*E* middle, duration post: genotype (*P* = 0.049), Kruskal–Wallis test, *n* = 102, 93, and 77 units; WT vs. HET (*P* = 0.01), WT vs. KO (*P* = 0.03), and HET vs. KO (*P* = 0.36), post hoc Mann–Whitney *U* test]. These results suggest that perceptual experience might have led to the circuit-level changes in SERT mice.

### Reduced Orientation and Oscillation Selectivity in SERT KO Mice After the Perceptual Experience

**Figure 2:**
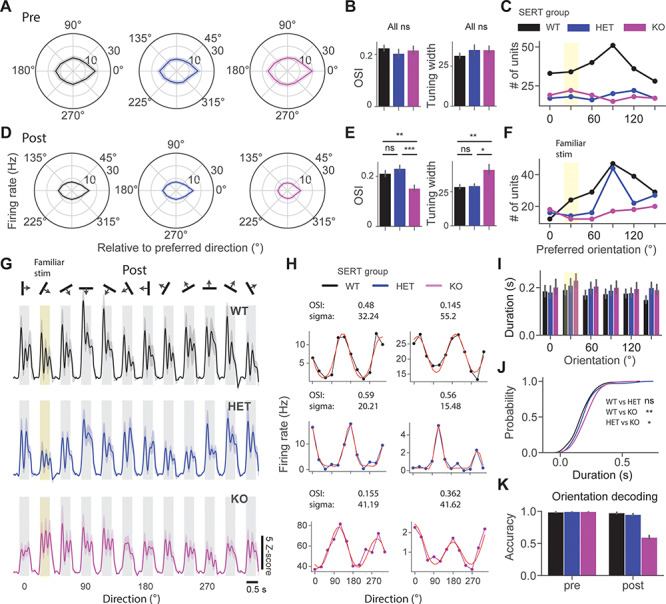
Reduced orientation and oscillation selectivity in SERT KO compared with WT and HET mice after the perceptual experience. (*A*) Polar plots show mean ± SEM of population direction tuning across 3 groups. (*B*) OSI and tuning width before perceptual experience. (*C*) The point plot shows the number of units preferring different orientations in naive mice. (*D*) Same as in (*A*), but after perceptual experience. (*E*) Same as in (*B*), but for experienced mice. (*F*) Same as in (*C*), but for experienced mice. (*G*) Averaged unit *z*-score responses to 12 different directions of 3 different groups. The 30° drifting grating was a familiar stimulus (highlighted in yellow). (*H*) Example direction tuning curves fitted with a double Gaussian (red) for the 3 different groups. (*I*) Bar plots show the mean ± SEM duration of oscillatory activity across different orientations. (*J*) Cumulative distribution function of the duration of oscillations across different groups. (*K*) Orientation decoding accuracy of classifiers trained on population spike counts from different groups using LDA.

To gain a deeper insight into the effects of perceptual experience on visual processing in SERT-deficient mice, we investigated cortical tuning properties. We first focused on orientation selectivity and tuning of V1 neurons. Sinusoidal drifting gratings of 12 different directions were presented to investigate orientation tuning properties. We first looked at population direction tuning curves of 3 different groups pre versus post perceptual experience (Fig. 2*A,D*). The polar plots were constructed by averaging direction tuning curves, which were aligned so that the preferred direction indicates 0°. We fitted a double Gaussian function to the direction tuning curves to quantify the tuning width, sigma (σ) (Fig. 2*H*). We did not find a significant difference in both OSI and tuning width between groups before perceptual experience [Fig. 2*B*, pre OSI: genotype (*P* = 0.82), Kruskal–Wallis test, *n* = 222, 106, and 110 units; sigma: genotype (*P* = 0.37), Kruskal–Wallis test, *n* = 196, 87, and 98 units]. Tuning width was comparable with what has been reported previously (Niell and Stryker 2008). We found an overrepresentation of preference for cardinal orientations in WT but not in other groups (Kreile et al. 2011) (Fig. 2*C*). Strikingly, we found a significantly lower OSI and wider tuning width in SERT KO compared with other groups after perceptual experience [Fig. 2*E*, post OSI: genotype (*P* = 0.0003), Kruskal–Wallis test, *n* = 172, 136, and 96 units, WT vs. HET (*P* = 0.05), WT vs. KO (*P* = 0.002), and HET vs. KO (*P* = 4.01E-5); post hoc Mann–Whitney *U* test; sigma: genotype (*P* = 0.02), Kruskal–Wallis test, *n* = 154, 122, and 72 units, WT vs. HET (*P* = 0.15), WT vs. KO (*P* = 0.004), and HET vs. KO (*P* = 0.02), post hoc Mann–Whitney *U* test]. We also found that both WT and HET had an overrepresentation of neurons preferring cardinal orientations after the perceptual experience (Fig. 2*F*).

We next investigated the oscillatory dynamics in response to drifting gratings. It has been previously shown that oscillations are partly specific to the orientation of the grating. We qualitatively observed that oscillations in SERT KO mice were evoked by a broad range of different directions (Fig. 2*G,I*). To compare oscillatory dynamics between groups, we averaged oscillatory duration across different stimuli for each unit. We found significantly longer oscillations in SERT KO mice compared with other groups [Fig. 2*J*, duration post: genotype (*P* = 0.001), Kruskal–Wallis test, *n* = 202, 134, and 109 units, WT vs. HET (*P* = 0.049), and WT vs. KO (*P* = 0.0002), and HET vs. KO (*P* = 0.023), post hoc Mann–Whitney *U* test]. Neural decoding analysis was then used to investigate whether reduced selectivity would affect orientation decoding. Using population spike counts pre versus post from different genotypes, we trained classifiers using LDA implemented in Python. We performed a 4-fold cross-validation with 5 repeats and found that orientation decoding accuracy dropped after perceptual experience only in SERT KO mice [Fig. 2*K*, orientation decoding accuracy mean ± standard error of the mean (SEM) % WT vs. HET vs. KO pre: (98.6 ± 0.3 vs. 99.4 ± 0.2 vs. 99.4 ± 0.2), *n* = 241, 116, and 120 units; post: (97.5 ± 0.6 vs. 94.9 ± 0.6 vs. 59.4 ± 1.6), *n* = 204, 148, and 109 units]. Together, our findings suggest that both orientation and oscillation selectivity were impaired in SERT KO but not in other groups following visual experience.

### Perceptual Experience Alters SF Processing in SERT KO Mice

**Figure 3:**
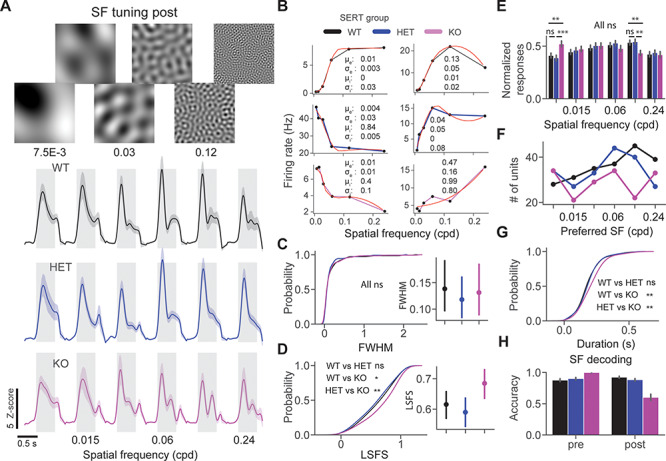
Spatial frequency nonspecific oscillations in SERT KO mice. (*A*) Average unit *z*-score firing rate in response to spatial frequency tuning stimuli across 3 different genotypes. (*B*) Example SF tuning curves fitted with a DoG. (*C*) Cumulative distribution function of FWHM of SF tuning curves. (*D*) The cumulative distribution function of LSFS, larger values indicate a lower attenuation at low SF. (*E*) Bar plots show the normalized responses across different SF and groups. (*F*) Point plots show the number of units preferring different SF. (*G*) Cumulative distribution function of oscillatory duration. (*H*) SF decoding accuracy of classifiers trained on population spike counts from different groups using LDA.

We next investigated SF tuning properties pre and post perceptual experience. Oscillations have been previously shown to be specific to the SF of the experienced stimulus. We designed visual stimuli of different spatial frequencies by performing spatial filtering of white noise in different frequency bands (Fig. 3*A*) to probe SF tuning and specificity of oscillations to the grating pattern. It has been previously shown that these stimuli can be used to probe SF tuning. We fitted a DoG model to SF tuning curves to quantify tuning bandwidth (Fig. 3*B*). We did not find any significant differences in the LSFS or the full width at half maximum (FWHM) of fitted SF tuning curves in naive mice (Supplementary Fig. 3). Inspection of average population responses revealed oscillatory activity at high SF only in SERT KO mice (Fig. 3*A*). We did not find a significant difference in the FWHM of the SF tuning curves between groups in experienced mice [Fig. 3*C*, FWHM post: genotype (*P* = 0.78), Kruskal–Wallis test, *n* = 145, 147, and 128 units], however, there was a significantly weaker suppression at low SF in SERT KO mice compared with other groups [Fig. 3*D*, LSFS post: genotype (*P* = 0.01), Kruskal–Wallis test, *n* = 171, 175, and 151 units, WT vs. HET (*P* = 0.21), WT vs. KO (*P* = 0.01), and HET vs. KO (0.002), post hoc Mann–Whitney *U* test]. Consistent with these findings, we discovered significantly different responses at the SF of 7.5E-3 and 0.12 cpd [normalized SF responses post: genotype SF = 7.5E-3 (*P* = 0.001), SF = 0.015 (*P* = 0.71), SF = 0.03 (*P* = 0.76), SF = 0.06 (*P* = 0.33), SF = 0.12 (*P* = 0.005), SF = 0.24 (*P* = 0.79), Kruskal–Wallis test, *n* = 215, 205, and 173 units]. Population responses were stronger at low and weaker at high SF in SERT KO [Fig. 3*E*, normalized SF responses post: SF = 7.5E-3: WT vs. HET (*P* = 0.1), WT vs. KO (*P* = 0.003), and HET vs. KO (0.0003), SF = 0.12: WT vs. HET (*P* = 0.44), WT vs. KO (*P* = 0.002), HET vs. KO (*P* = 0.002), post hoc Mann–Whitney *U* test, *n* = 215, 205, and 173 units]. Units preferring higher than trained SF (0.06 and 0.12 cpd) were overrepresented in both WT and HET but not in SERT KO mice (Fig. 3*F*).

We next quantified the duration of oscillatory activity in response to different SF stimuli. The duration of oscillations was averaged across stimuli for each unit and compared across different genotypes (Fig. 3*E*). Significantly longer oscillations were found in SERT KO mice compared with the 2 other groups [Fig. 3*G*, duration post: genotype (*P* = 0.016), Kruskal–Wallis test, *n* = 208, 198, and 156 units, WT vs. HET (*P* = 0.26), WT vs. KO (*P* = 0.003), and HET vs. KO (*P* = 0.011), post hoc Mann–Whitney *U* test]. We then investigated SF decoding pre versus post visual learning. Classifiers were trained for different conditions on population spike counts using LDA in scikit-learn. SF decoding accuracy was reduced after learning in SERT KO but not in other groups [Fig. 3*H*, SF decoding accuracy mean ± SEM % WT vs. HET vs. KO: pre (87.8 ± 1.1 vs. 90.2 ± 1.1 vs. 99.8 ± 0.2), *n* = 241, 140, and 197 units; post (92.5 ± 0.8 vs. 88.3 ± 1.0 vs. 60.2 ± 2.5), *n* = 229, 209, and 174 units]. Overall, our results suggest that there is altered SF processing in SERT KO after the perceptual experience.

### Lower Contrast Sensitivity After Perceptual Experience in SERT HET Mice

**Figure 4:**
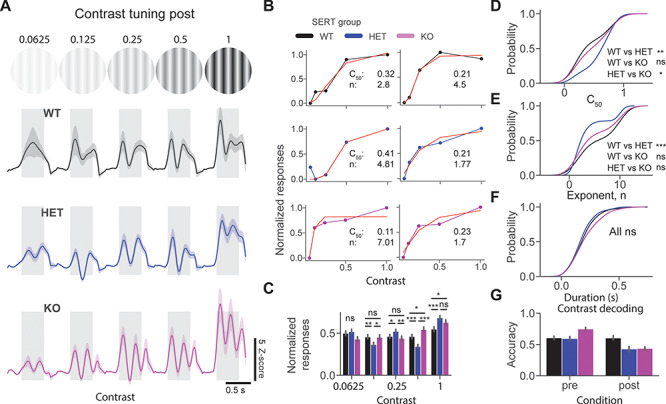
Lower contrast sensitivity after perceptual experience in SERT HET mice. (*A*) Average population *z*-score firing rate in response to a grating stimulus at various contrast levels in 3 different groups. (*B*) Example contrast response curves fitted with a ratio of hyperbolic function from 3 different groups. (*C*) The bar plot shows the mean normalized response to the visual stimulus at different contrast levels. (*D*) Cumulative distribution function of *c*_50_ (lower values indicate higher contrast sensitivity) across different genotypes. (*E*) The cumulative distribution function of the “*n*” (exponent) parameter of the fitted curve. (*F*) The cumulative distribution function of oscillatory duration across different genotypes. (*G*) Contrast decoding accuracy of classifiers trained on population spike counts from different genotypes using LDA.

We next focused on contrast sensitivity properties of 3 genotypes pre and post perceptual experience. No significant alterations were found in contrast response function in naive SERT-deficient mice (Supplementary Fig. 4). We then measured the contrast sensitivity after visual experience. Mean population *z*-score responses to 0° static grating at different contrast levels revealed oscillatory activity only in SERT KO mice. Normalized responses were differentially modulated by contrast in different groups [Fig. 4*C*, normalized responses at different contrast levels post: genotype C = 0.0625 genotype (*P* = 0.05); C = 0.125 genotype (*P* = 0.016), WT vs. HET (*P* = 0.004), WT vs. KO (*P* = 0.41), and HET vs. KO (*P* = 0.01); C = 0.25 genotype (*P* = 0.03), WT vs. HET (*P* = 0.02), WT vs. KO (*P* = 0.24), and HET vs. KO (*P* = 0.006); C = 0.5 genotype (*P* = 1.56E-6), WT vs. HET (*P* = 0.0008), WT vs. KO (0.02), and HET vs. KO (*P* = 1.11E-7); C = 1.0 genotype (*P* = 0.003), WT vs. HET (*P* = 0.0003), WT vs. KO (*P* = 0.04), and HET vs. KO (*P* = 0.05), Kruskal–Wallis test with post hoc Mann–Whitney *U* test, *n* = 209, 197, and 171 units]. We fitted hyperbolic ratio functions to the contrast sensitivity curves (Fig. 4*B*). Significantly lower contrast sensitivity (higher *c*_50_ values) was found in HET mice [Fig. 4*D*, *c*_50_ post: genotype (*P* = 0.01), Kruskal–Wallis test, *n* = 74, 78, and 75 units, WT vs. HET (*P* = 0.001), and WT vs. KO (*P* = 0.25), and HET vs. KO (*P* = 0.01), post hoc Mann–Whitney *U* test]. Furthermore, the exponent (“*n*” parameter) was significantly lower in HET versus WT [Fig. 4*E*, exponent post: genotype (*P* = 0.008), Kruskal–Wallis test, *n* = 74, 78, and 75 units, WT vs. HET (*P* = 0.0008), WT vs. KO (*P* = 0.07), and HET vs. KO (*P* = 0.05), post hoc Mann–Whitney *U* test]. Despite qualitative differences in oscillatory dynamics, we did not find significant differences in duration of oscillations between genotypes [Fig. 4*F*, duration post: genotype (*P* = 0.14), Kruskal–Wallis test, *n* = 165, 151, and 137 units]. Using populations spike counts within 0.05–0.5 s of the stimulus onset, we trained classifiers for different conditions to decode the contrast of the presented stimulus. We saw an overall decrease in the performance of the classifiers in SERT-deficient mice after perceptual experience [Fig. 4*G*, contrast decoding accuracy mean ± SEM % WT vs. HET vs. KO: pre (60.6 ± 1.6 vs. 59.4 ± 1.7 vs. 74.8 ± 1.3), *n* = 239, 137, and 189 units; post (60.4 ± 1.8 vs. 42.8 ± 2.2 vs. 43.6 ± 1.3), *n* = 211, 203, and 175 units]. Together, our results suggest that contrast sensitivity is altered in SERT-deficient mice after perceptual experience, especially in HET.

## Discussion

Here we investigated visual processing and experience-dependent learning in SERT-deficient mice (Table 1). We did not find significant alterations in orientation, SF, and contrast tuning in naive mice. This finding is aligned with the prior operant conditioning study that demonstrated intact learning in visual discrimination tasks in SERT-deficient mice (Brigman et al. 2009). Furthermore, compensatory mechanisms might partially correct for the lack of functional SERT to maintain cortical development (Zhou et al. 2002). However, we observed a lack of bias towards cardinal orientations in V1 of SERT-deficient mice before visual experience. It was partially recovered in SERT HET mice after the perceptual experience but not in KO animals.

**Table 1:** Summary of alterations in SERT-deficient mice compared with WT

Condition	Alteration	HET	KO
Pre	Stimulus-evoked theta	↑	↑
	Stimulus-evoked beta	↓	↓
	Stimulus-evoked low gamma	↑	↑
	Population unit responses	NS	↑
	Bias towards cardinal orientations	↓	↓
	Responses to low contrast stimuli	NS	↓
	Responses to medium contrast stimuli	↑	↑
Post	Duration of unit oscillatory activity	↑	↑
	OSI	NS	↓
	Bias towards cardinal orientations	NS	↓
	Orientation decoding accuracy	NS	↓
	LSFS	NS	↑
	SF decoding accuracy	NS	↓
	*c*_50_	↑	NS
	Contrast decoding accuracy	↓	↓
Novel	Stimulus-evoked population unit responses	NS	↓
	Pupillary surprise response	NS	↓
	Stimulus-evoked alpha	↓	↓
	ITPC in low-frequency oscillations	↓	↓

Note: Table showing a summary of changes found in SERT-deficient mice compared with WT across different conditions. (↑) indicates stronger/longer, (↓) weaker/shorter, (NS) not significant, (pre) naive mice, (post) after the perceptual experience, and (novel) in response to a novel stimulus.

Visual experience-dependent oscillations can be used as a global proxy for plasticity. We have previously shown that perceptual experience induces theta oscillations in both LFP and single-unit activity (Kissinger et al. 2018). Furthermore, these oscillations were weaker in the Fmr1 KO mouse model of autism (Kissinger et al. 2020). Our observations of longer oscillatory activity in SERT KO mice might indicate enhanced plasticity. It has been previously shown that serotonin signaling might be important for long-term depression and regulation of excitatory–inhibitory balance through various 5-HT receptors in cortical neurons (William Moreau et al. 2009; He et al. 2015; Berthoux et al. 2019). We also observed reduced feature specificity of these oscillations in SERT KO mice. Such impairments might arise from cross-feature activation and nonspecific tuning in the visual cortex after the perceptual experience.

Perceptual learning was shown to improve cortical tuning in the visual cortex (Jurjut et al. 2017). Furthermore, altered visual experience during development was shown to affect cortical orientation preference (Kreile et al. 2011). Fine-tuning of cortical circuits during adulthood is limited. However, a SERT inhibitor, fluoxetine, was shown to be able to restore critical period in adult rats’ visual cortex, which supports the role of 5-HT in adult plasticity (Maya Vetencourt et al. 2008). Our observations of longer oscillatory activity after perceptual experience in SERT-deficient mice further support the theory that 5-HT may play a role in cortical plasticity. We also observed a decreased orientation selectivity and broadened orientation tuning in SERT KO mice. Interestingly, decreased orientation selectivity and broadened orientation tuning are similar to the alterations in Fmr1 KO mice, which are known to be mediated by the hypoactivation of parvalbumin-positive fast spiking interneurons and their corresponding circuit alterations (Goel et al. 2018; Kissinger et al. 2020). Thus, one of the potential mechanisms underlying similar alterations in SERT KO mice may be mediated by the impaired plasticity of the cortical inhibitory circuitry following visual experience. SF and contrast processing were also impaired after perceptual learning. Although fluoxetine was shown to improve visual acuity in adult rats, recent clinical trials in humans report diverse findings regarding the efficacy of the antidepressants in managing amblyopia (Huttunen et al. 2018; Lagas et al. 2019; Sharif et al. 2019). Thus, it is possible that 5-HT-mediated enhanced plasticity is not sufficient for functional recovery of vision in humans, and additional pharmacological or perceptual training protocols may also be required.

Our pupillometry recordings did not reveal significant alterations in SERT-deficient mice. Similarly to our previous study, we observed a strong surprise response in pre and novel, but not in the familiar condition (Kissinger et al. 2018). There was a trend towards increased pupil size in SERT KO mice, but it was not significant. We observed a smaller surprise response to the novel stimulus in SERT KO, which might arise from decreased sensitivity to novelty. Reduced novelty response might be associated with a reduced specificity of theta oscillations and activity of the noradrenergic system. Alternatively, a large baseline pupil size might limit the dynamic range of pupil dynamics in SERT KO mice.

It is important to note that transgenic animals lacking a functional SERT variant might display various deficits beyond the serotonergic system. They might arise, in part, due to the compensatory mechanisms during the development. Therefore, it would be important for future studies to use specific 5-HT receptor agonists/antagonists or transient pharmacological/optogenetic manipulations to establish a direct link between serotonin signaling and visual processing and learning.

The major findings of our study were related to impaired tuning after visual learning. Given that we observed largely intact tuning properties in single units in naive SERT mice, similar oscillatory dynamics in LFP after visual learning in all groups and similar pupil responses to familiar stimuli, it is likely that altered serotonin signaling is one of the major factors underlying our observations.

In conclusion, we provide evidence for the impaired fine-tuning of cortical selectivity and longer familiarity-evoked theta-locked spiking activity following perceptual experience in V1 of SERT-deficient mice. Our findings suggest that 5-HT signaling may be involved in the experience-dependent refinement of cortical circuitry and encoding of visual familiarity in mice. Future studies will dissect the molecular and circuit mechanisms of this signaling in V1 cortical plasticity.

## Notes

A.P. and A.A.C. designed the study, A.P. performed the experiments and analyzed the data, and A.P. and A.A.C. wrote the manuscript.

The authors thank Dr Sotiris Masmanidis for the provided silicon probes. *Conflict of Interest*: None declared.

## Funding

This work was supported by Whitehall Foundation Research Grant, Purdue Research Foundation and National Institute of Mental Health Grant R01 MH116500.

## Supplementary Material

Suppl_Fig1_tgaa066

Suppl_Fig2_tgaa066

Suppl_Fig3_tgaa066

Suppl_Fig4_tgaa066

Suppl_Fig5_tgaa066

SERT_paper_supplementary_final_tgaa066
