# Supplementary figures and images for: Cortical Tuning is Impaired After Perceptual Experience in Primary Visual Cortex of Serotonin Transporter-Deficient Mice
Source: Cereb Cortex Commun. 2020 Sep 16;1(1):tgaa066. doi: 10.1093/texcom/tgaa066 (PMC7575641; doi:10.1093/texcom/tgaa066)

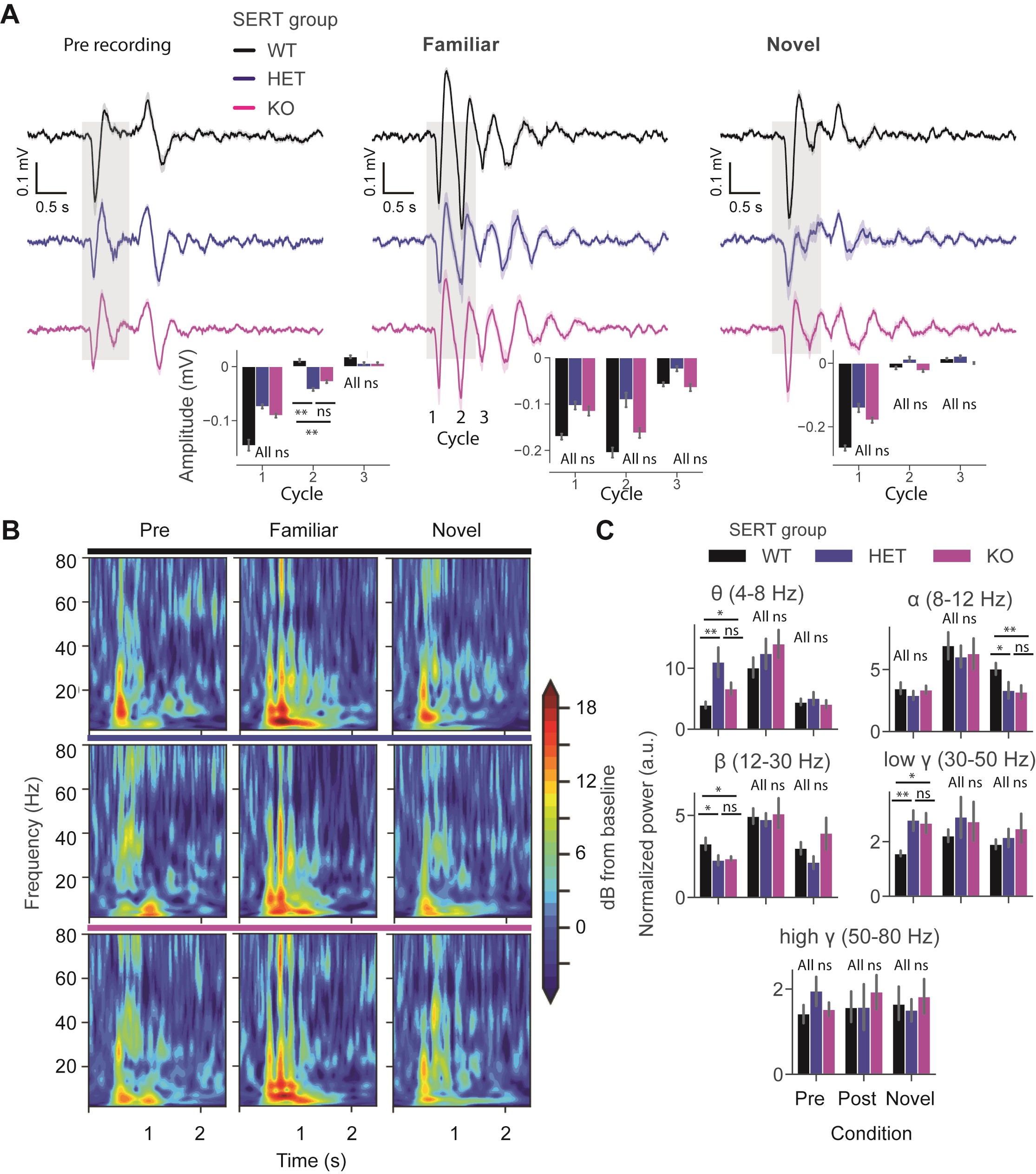

Supplement: Suppl_Fig1_tgaa066 [file suppl_fig1_tgaa066.jpeg]

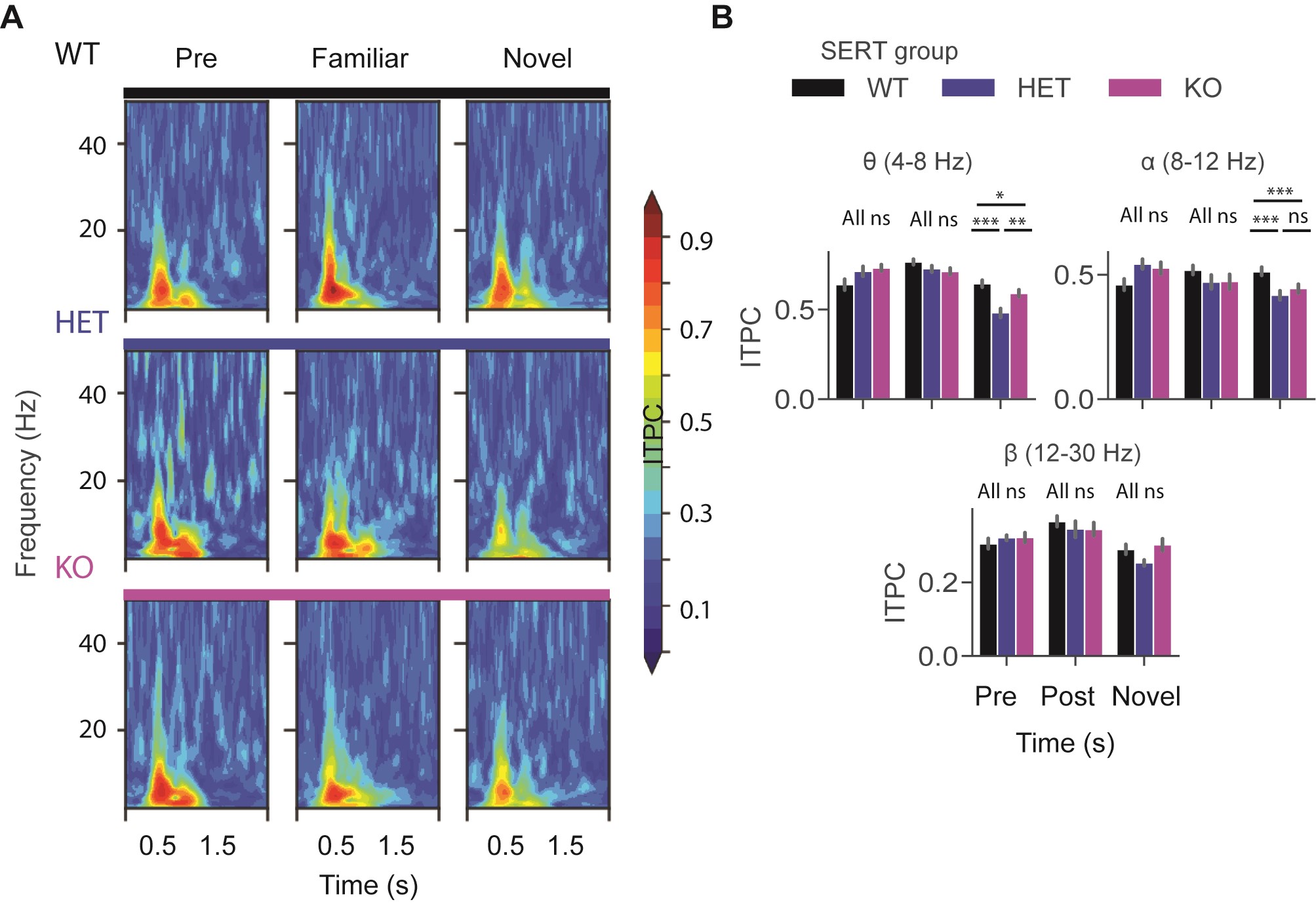

Supplement: Suppl_Fig2_tgaa066 [file suppl_fig2_tgaa066.jpeg]

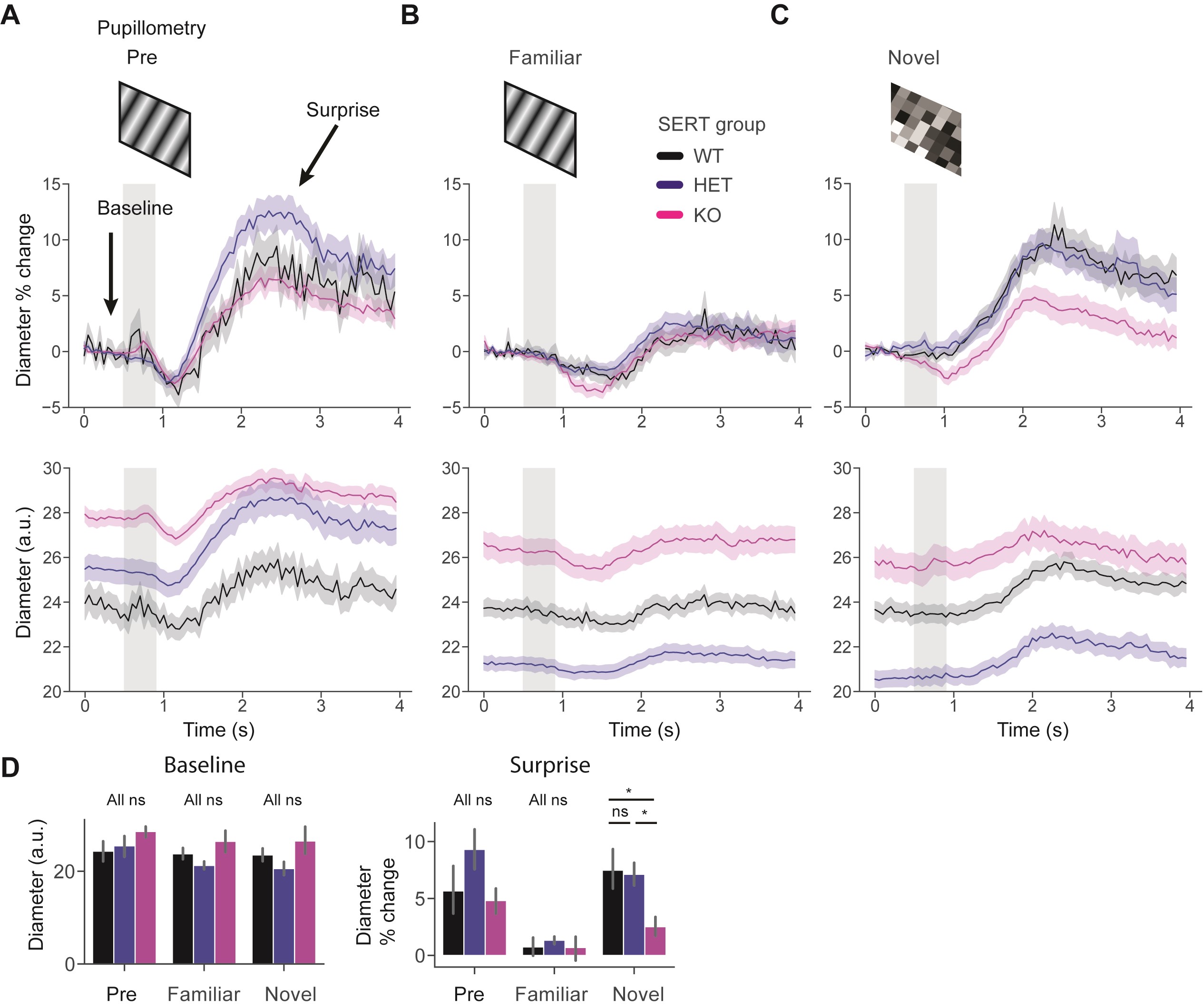

Supplement: Suppl_Fig3_tgaa066 [file suppl_fig3_tgaa066.jpeg]

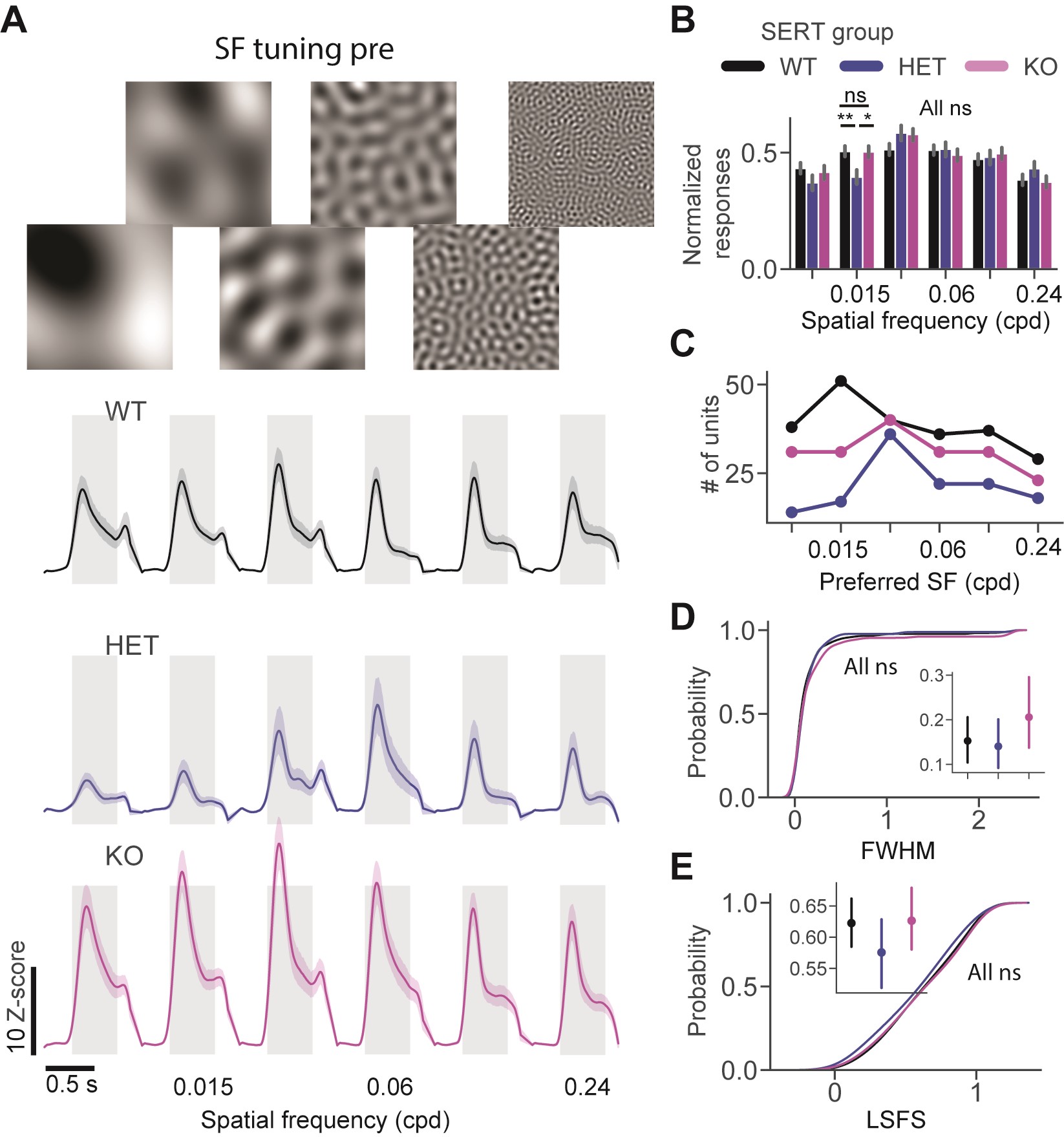

Supplement: Suppl_Fig4_tgaa066 [file suppl_fig4_tgaa066.jpeg]

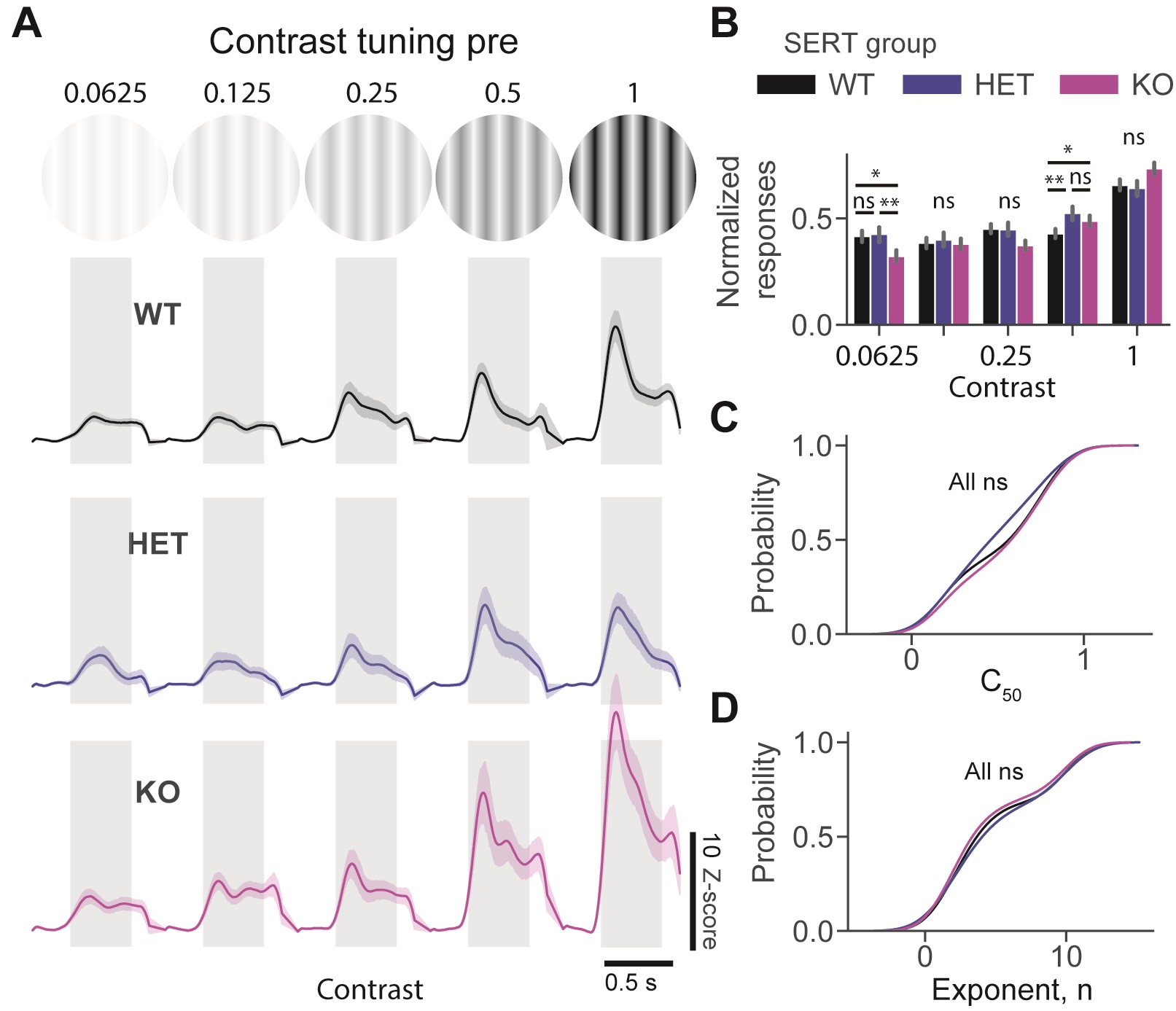

Supplement: Suppl_Fig5_tgaa066 [file suppl_fig5_tgaa066.jpeg]
