# Supplementary material for: Cortical Tuning is Impaired After Perceptual Experience in Primary Visual Cortex of Serotonin Transporter-Deficient Mice
Source: Cereb Cortex Commun. 2020 Sep 16;1(1):tgaa066. doi: 10.1093/texcom/tgaa066 (PMC7575641; doi:10.1093/texcom/tgaa066)

**Supplementary Figure 1.**Altered oscillatory dynamics pre but not after visual experience in SERT deficient mice. **A.** Averaged layer 4 LFP traces of SERT WT, HET, and KO in different conditions: pre (left), post familiar (middle), and post novel (right). Inset bar plots show the mean ± s.e.m. of the amplitude (pre: cycle1 (P = 0.49), cycle2 (P = 0.01), and cycle3 (P = 0.46), Kruskal-Wallis test, n = 7, 6, and 9 mice; post hoc cycle2: WT vs HET (P = 0.006), WT vs KO (P = 0.009), and HET vs KO (P = 0.46), Mann-Whitney U test; post: cycle1 (P = 0.35), cycle2 (P = 0.35), and cycle3 (P = 0.37), Kruskal-Wallis test, n = 7, 8, and 9 mice; novel: cycle1 (P = 0.27), cycle2 (P = 0.59), and cycle3 (P = 0.27), Kruskal-Wallis test, n = 7, 8, and 9 mice). **B.** Time frequency spectra of LFP traces of WT (top), HET (middle), and KO (bottom). **C.** Bar plots show the mean ± s.e.m. of normalized power across different frequency bands (pre: $\theta$ (P = 0.02), $\alpha$ (P = 0.66), $\beta$ (P = 0.10), low $\gamma$ (P = 0.02), and high $\gamma$ (P = 0.47), Kruskal-Wallis test, n = 7, 6, and 9 mice, post hoc $\theta$ WT vs HET (P = 0.006), WT vs KO (P = 0.028), HET vs KO (P = 0.131), low $\gamma$ WT vs HET (P = 0.004), WT vs KO (P =0.022), and HET vs KO (P = 0.476), Mann-Whitney U test; post: $\theta$ (P = 0.54), $\alpha$ (P = 0.68), $\beta$ (P = 0.91), low $\gamma$ (P = 0.96), and high $\gamma$ (P = 0.49), Kruskal-Wallis test, n = 7, 8, and 9 mice; novel: $\theta$ (P = 0.84), $\alpha$ (P = 0.05), $\beta$ (P = 0.31), low $\gamma$ (P = 0.85), and high $\gamma$ (P = 0.97), Kruskal-Wallis test, n = 7, 6, and 9 mice).


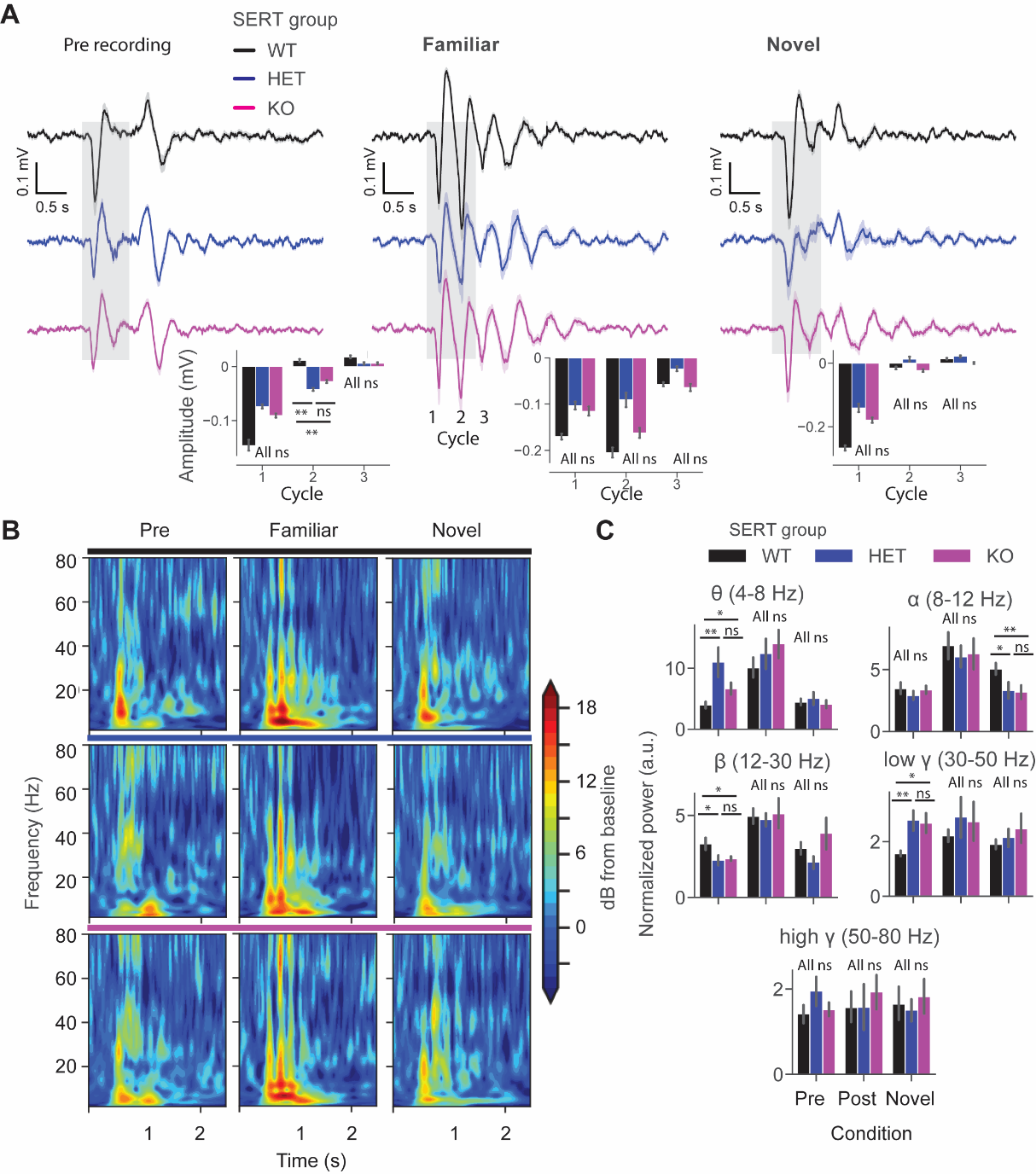


**Supplementary Figure 2.** Inter-trial phase coherence (ITPC) is lower in response to the novel stimulus in SERT HET and KO vs WT mice in low frequency bands. **A.** Heatmaps show ITPC of the LFP traces of WT (top), HET (middle), and KO (bottom) for pre (left), post visual experience (middle), and novel stimulus (right). **C.** Bar plots show the mean ± s.e.m. of mean ITPC within 0-0.5 s relative to the stimulus onset across different frequency bands (**pre**: $\theta$ (P = 0.302), $\alpha$ (P = 0.104), $\beta$ (P = 0.521), Kruskal-Wallis test, n = 18, 6, and 18 channels; **post**: $\theta$ (P = 0.071), $\alpha$ (P = 0.387), $\beta$ (P = 0.483), Kruskal-Wallis test, n = 21, 12, and 24 channels; **novel**: $\theta$ (P = 0.0001), $\alpha$ (P = 0.0001), $\beta$ (P = 0.115),, Kruskal-Wallis test, n = 18, 12, and 19 channels; post hoc $\theta$ WT vs HET (P = 3.8e-5), WT vs KO (P = 0.048), and HET vs KO (P = 0.0016); $\alpha$ WT vs HET (P = 0.0001), WT vs KO (P = 0.0003), and HET vs KO (P = 0.150), Mann-Whitney U test).


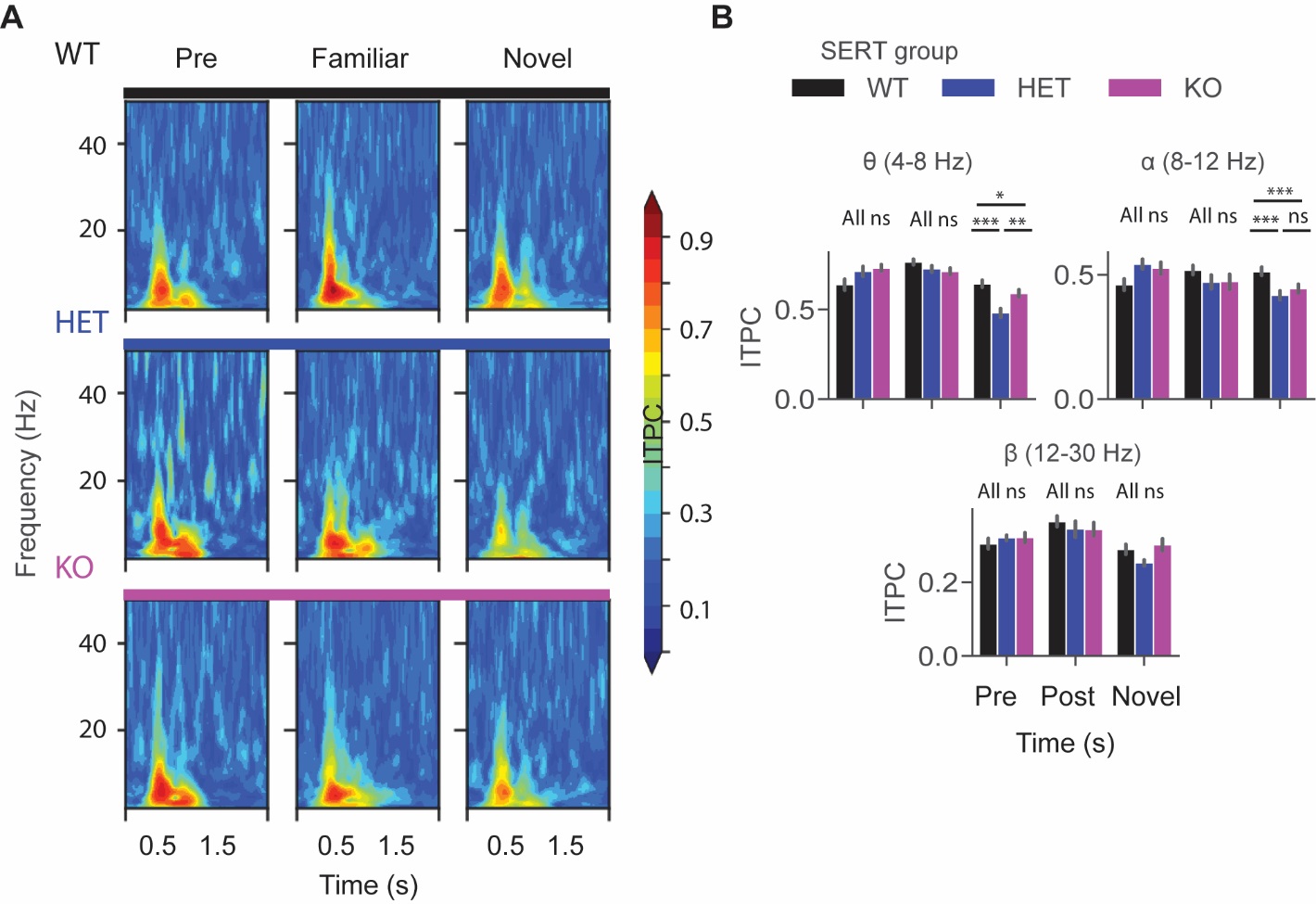


**Supplementary Figure 3.** Surprise response in SERT KO mice. **A.** Normalized (top) and raw (bottom) pupil diameter in response to drifting grating stimulus in naive mice. Note: strong surprise response due to the novelty of the stimulus. **B.** Same as in A, but after perceptual experience to the familiar stimulus. Note: surprise response is small due to the familiarity to the stimulus. **C.** Same as in B, but for the novel stimulus. Note: strong surprise response due to the novelty of the stimulus. **D.** Bar plots show the mean ± s.e.m. of raw diameter (left) for baseline pupil size or normalized diameter (right) for surprise response across different conditions. (Baseline pupil size: pre (P = 0.24), post (P = 0.21), and novel (P = 0.32), Kruskal-Wallis test, n = 7 WT, 6 HET, and 6 KO mice; surprise: pre (P = 0.15), post (P = 0.68), novel (P = 0.03), Kruskal-Wallis test, n = 7 WT, 6 HET, and 6 KO mice , post hoc novel WT vs HET (P = 0.47), WT vs KO (P = 0.02), and HET vs KO (P = 0.01), Mann-Whitney U test).


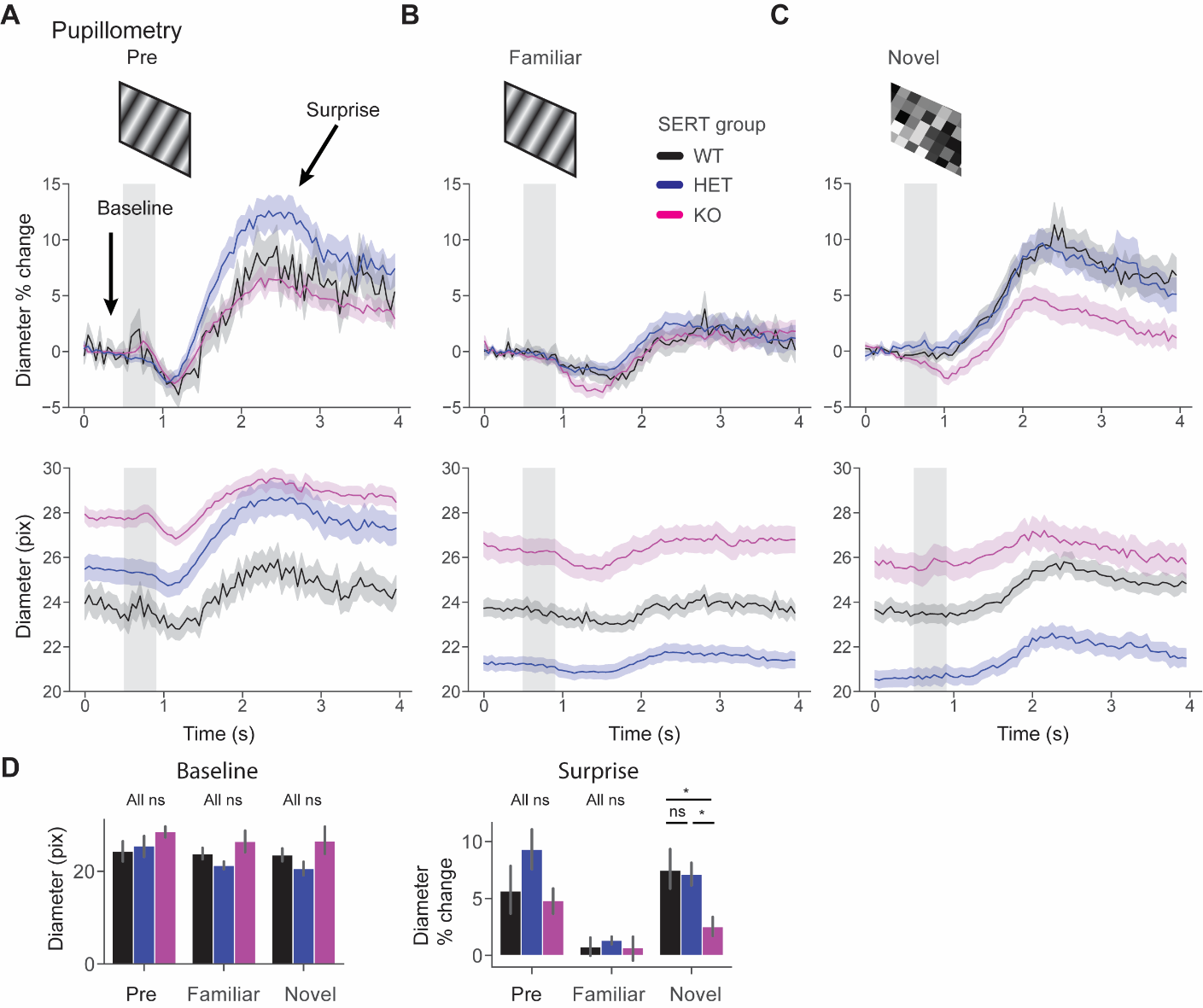


**Supplementary Figure 4.** Intact spatial frequency tuning in naive SERT mice but weaker responses to low SF in SERT HET. **A.** Average unit z-score firing rate in response to spatial frequency tuning stimuli. **B.** Bar plots show the normalized responses across different SF and groups (SF=7.5E-3 (P = 0.31), SF = 0.015 (P = 0.009), SF = 0.03 (P = 0.07), SF = 0.06 (P = 0.74), SF = 0.12 (P =0.77), and SF = 0.24 (P = 0.27), Kruskal-Wallis test, n = 231, 129, and 187, post hoc SF = 0.015 WT vs HET (P = 0.002), WT vs KO (P = 0.42), and HET vs KO (P = 0.004), Mann-Whitney U test) **C.** Point plots how the number of units preferring different SF. **D.** Cumulative distribution function of FWHM of SF tuning curves across three different genotypes (FWHM pre (P = 0.47), Kruskal-Wallis test, n = 177, 90, and 130 units). **E.** Cumulative distribution function of LSFS across three different groups (LSFS pre (P = 0.34), Kruskal-Wallis test, n = 200,m 111, and 140 units).


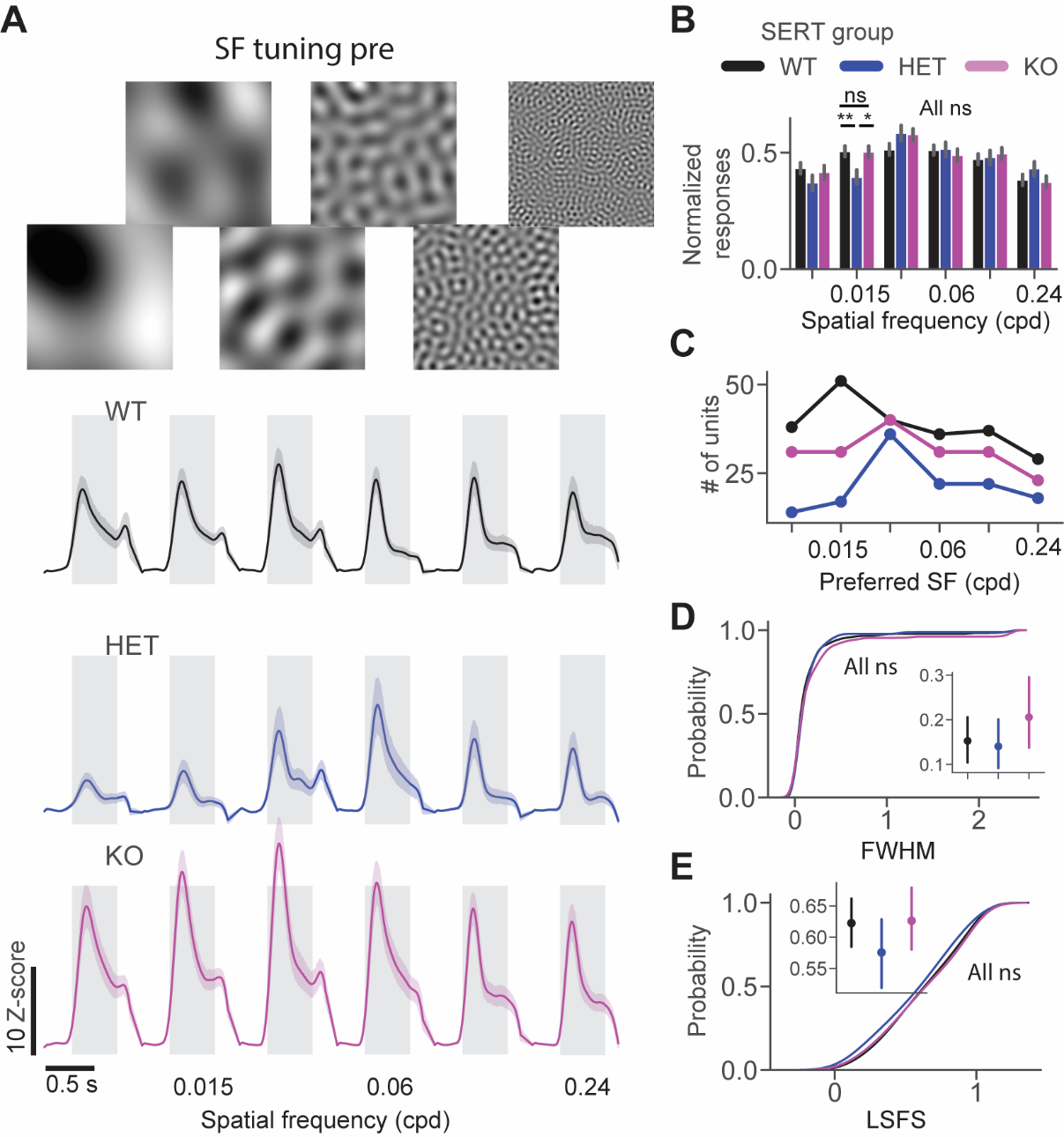


**Supplementary Figure 5.** Weaker responses to low contrast stimuli in SERT KO and stronger responses to medium contrast stimuli in naive SERT deficient vs WT mice. **A.** Average population z-score firing rate in response to the grating stimulus at various contrast levels in three different groups. **B.** Bar plot shows the mean normalized response to the visual stimulus at different contrast levels (C=0.0625 (P = 0.025), C = 0.125 (P = 0.891), C = 0.25 (P = 0.05), C = 0.5 (P = 0.03), C = 1.0 (P =0.05), Kruskal-Wallis test, n = 228, 131, and 183, post hoc C = 0.0625 WT vs HET (P = 0.41), WT vs KO (P = 0.01), and HET vs KO (P = 0.008), C = 0.5 WT vs HET (P = 0.008), WT vs KO (P = 0.03), and HET vs KO (P = 0.21), Mann-Whitney U test). **C.** Cumulative distribution function of c_50_ (lower values indicate higher contrast sensitivity) across different genotypes (c_50_ pre (P = 0.41), Kruskal-Wallis test, n = 109, 58, and 107 units). **D.** Cumulative distribution function of the “n” (exponent) parameter of the fitted curve (exponent pre (P = 0.58), Kruskal-Wallis test, n = 109, 58, and 107 units).


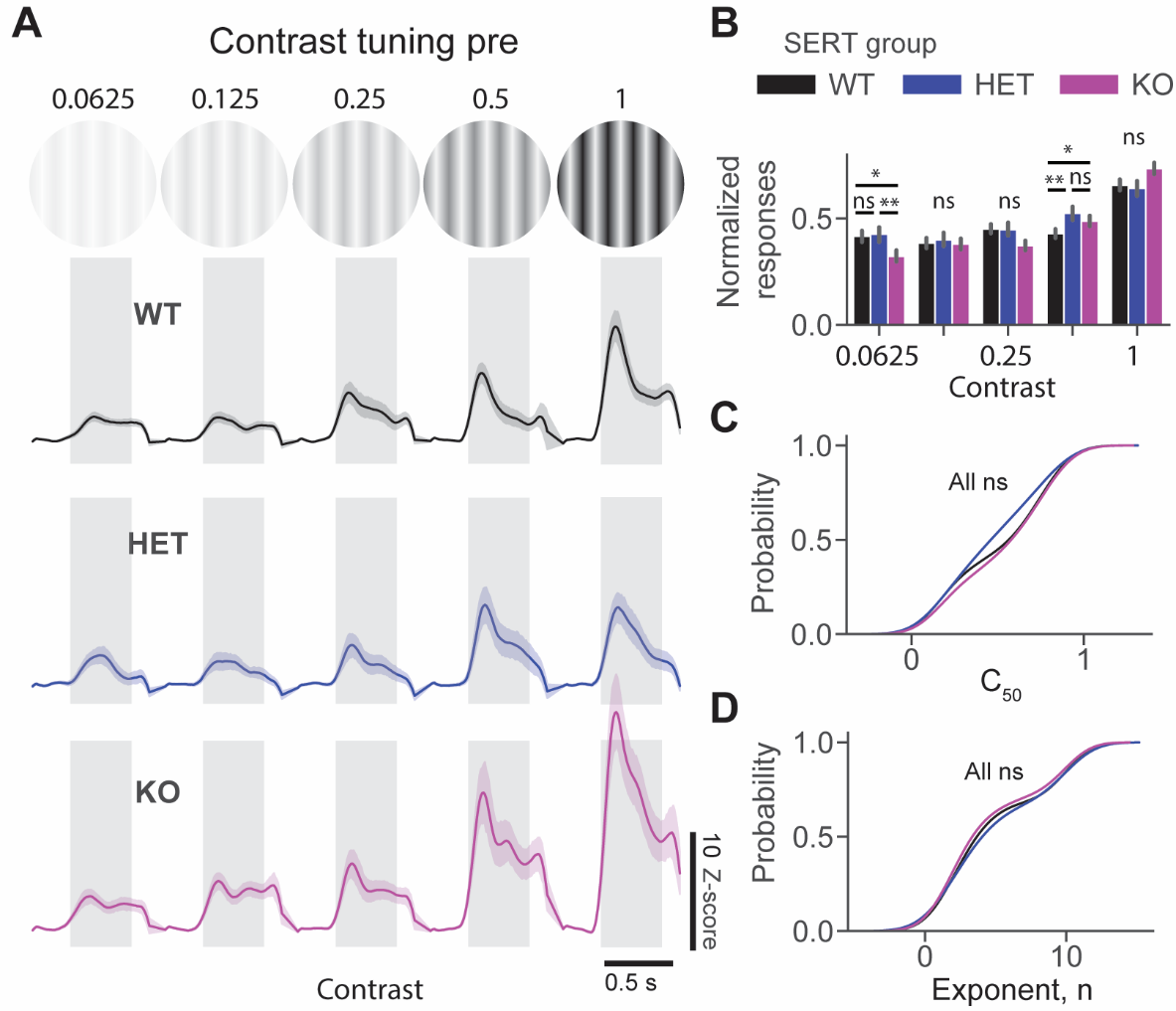

Supplement: SERT_paper_supplementary_final_tgaa066 [file sert_paper_supplementary_final_tgaa066.docx]
